# Opposite effects of non-thermal plasma on cell migration and collagen production in keloid and normal fibroblasts

**DOI:** 10.1371/journal.pone.0187978

**Published:** 2017-11-16

**Authors:** Sung Un Kang, Yeon Soo Kim, Yang Eun Kim, Ju-Kyeong Park, Yun Sang Lee, Hee Young Kang, Jae Won Jang, Jeong Beom Ryeo, Yuijina Lee, Yoo Seob Shin, Chul-Ho Kim

**Affiliations:** 1 Department of Otolaryngology, Ajou University, Suwon, Korea; 2 Department of Molecular Science and Technology, Ajou University, Suwon, Korea; 3 Department of Otorhinolaryngology, College of Medicine, Konyang University Hospital, Konyang University Myunggok Medical research Institute, Daejeon, Korea; 4 Department of Dermatology, Ajou University, Suwon, Korea; 5 Department of Otorhinolaryngology, Chungnam National University, Daejeon, Korea; 6 ICD co., LTD, Anseong, Korea; Universite Toulouse III Paul Sabatier, FRANCE

## Abstract

Recent progress in the understanding non-thermal plasma (NTP) properties prompted its application in the treatment of various diseases. However, therapeutic effect of NTP on keloid cells has not been reported previously. We sought to investigate the effect of NTP treatment on keloid by comparing cell migration and collagen production of keloid (KFs) and normal fibroblasts (NFs) and determined the regulatory pathways involved. We assessed NTP effects on cell migration in KFs and NFs by the wound healing assay and measured the expression of the epidermal growth factor receptor (EGFR), signal transducer and activator of transcription-3 (STAT3), and collagen by western blot. Expression of the transforming growth factor-β and Type I collagen following NTP treatment was determined by reverse transcription-polymerase chain reaction, immunofluorescence staining, and the Sircol collagen assay. NTP treatment increased cell migration and collagen production of NFs. However, it reduced these parameters in KFs. NTP reduced the expression of EGFR, STAT3, and Type I collagen in KFs but increased their levels in NFs. We revealed that NTP suppressed KF cell migration via down-regulation of EGFR and STAT3 and reduced collagen production via supressing transforming growth factor-β. Our data suggest that NTP may be a new therapeutic strategy for keloids.

## Introduction

Keloid is a poorly regressing cutaneous lesion characterised by haphazard dermal fibrosis and excessive skin growth beyond original wound boundaries, which reflects pathological wound healing [1]. Keloid scars (KS) are cosmetically frustrating problems frequently associated with functional deformities. Keloid treatment strategies include steroid injections, radiotherapy, cryotherapy and excisional surgery, but they do not provide satisfactory outcomes [2–4]. Although keloid pathophysiology is still unclear, there are several molecular mechanisms known to be involved, including signalling regulated by growth factors such as epidermal growth factor receptor (EGFR) and vascular endothelial growth factor [5–7]. Keloid fibroblasts (KFs) produce about 20 times more collagen than normal fibroblasts (NFs) due to increased collagen synthesis [8]. Previous studies have demonstrated that keloid formation occurs in response to the transforming growth factor-β (TGF-β) stimulation [9, 10]. Recent study has proved that the TGF-β induces Type I collagen overexpression in keloid scarring [1]. Probing molecular signalling events in keloid are necessary to understand its pathophysiology and to discover new therapeutic options [6].

In recent years, there has been an increased interest in properties of the non-thermal plasma (NTP) as a novel therapeutic method. Plasma medicine, which involves medical use of partially ionized gas, which comprises electrons, ions, radicals, and energetic photons, is a rapidly growing field that may potentially unlock novel treatment modalities [11]. NTP has been recently used for several indications including infections, wound healing, blood coagulation, and cancer [12–15]. Our group previously showed that NTP suppresses cell migration, induces cell growth arrest and delays tumor invasion in cancer models [12, 16]. It is also reported that mild NTP inactivates microorganisms and improves wound healing via increased integrin expression and modulating adhesion molecules and matrix metalloproteinase-9 [17, 18]. However, there are no studies that demonstrated the effectiveness of NTP on keloid treatment until now. Because of the tumor-suppressive ability of NTP, we postulated that NTP could reduce skin defects associated with keloids which is hypercellular lesion. In this study, we found that NTP treatment suppressed cell migration of KFs in vitro, but it enhanced cell migration of NFs and determined that the inhibitory mechanism of NTP on the cell migration and collagen production in KF is mediated by regulation of signal pathways.

## Materials and methods

### Cell culture

5 mm sized KS and normal skin (NS) tissues were extracted from 10 patients (KS, n = 5, 2 men and 3 women and NS, n = 5, 2 men and 3 women as a mean age of 25 years each), who gave written informed consent for use of their excised tissue for academic research. The NF and KF that we used in this study were established by primary culture from human NS and KS. The institutional review board of Ajou University School of Medicine approved this study (AJIRB-GEN-GEN-12-107). The lesions were diagnosed as keloids on the basis of clinical appearance, persistence for more than 1 year, and extension beyond the original wound boundaries. Normal tissue specimens were obtained from healthy adult foreskins. KFs and NFs were maintained in the RPMI-1640 medium supplemented with 10% heat-inactivated foetal bovine serum and 1% antibiotic/antimycotic solution (all from Gibco/BRL, Grand Island, NY, USA). All cultures were maintained at 37°C in humidified (5% CO_2_) incubators. Cells were sub-cultured at 80–90% confluence using trypsin. Only cells from passages two to seven were analysed in this study.

### Experimental system specification

Dielectric barrier discharge was utilised in an atmospheric pressure plasma system (S1 Fig), which uses quartz or ceramic tube, and has a multi-nozzle NTP source. The gas supply dielectric tube nozzle diameter is less than 3mm and is designed for 1 inch sized uniform plasma jet used for biomedical research applications. This system is highly effective for the surface modification of biological samples at low temperatures, which is critical for biological experiments.

To produce plasma at atmospheric pressure, a high voltage low frequency electric power module with a pulse width modulation controller integrated circuit was utilised to set the operation frequency of 25 kHz. The power supply for this system used a high voltage transformer (1:100) to generate 3 kV peak, and these specifications can vary with the type and amount of gas used. In this study, argon (Ar) and nitrogen (N_2_) were used as carrier gases and the ratio of Ar/N_2_ is 200:1500. The distance between the multi-nozzles and the sample were 2 cm.

### Wound healing assay

Cells were plated in 12-well culture plates at a density of approximately 5×10^5^ / well and grown to confluence. The wound healing assay was performed as described previously [19]. In brief, the cell monolayer was scratched with a sterile pipette tip, followed by extensive washing to remove cellular debris. The cells were then exposed to N_2_/Ar NTP or gas only for 10 s and 30 s, respectively.

### Annexin-V and propidium iodide staining

Cell death was detected using a FITC (Fluorescein isothiocyanate) Annexin V-PI (propidium iodide) apoptosis detection kit I, according to the manufacturer’s protocol (BD Biosciences, Bedford, MA, USA) as described previously [20].

### Cell viability assay

Cells were seeded in 96-well plates at a density of 5×10^3^ cells/well, incubated for 24 and 48 hours. Cell viability after the treatment with N_2_/Ar NTP for various periods (0, 10, 30 or 60 s) was measured using an assay based on the conversion of MTT (3-(4,5-dimethylthiazol-2-yl)-2,5-diphenyl-tetrazoliumbromide; Sigma–Aldrich, St. Louis, MO, USA). Cell viability assays were performed as described previously [21]. The optical density of each culture well was measured using a microplate reader (Bio-Tek, Winooski, VT, USA) at 540 nm. Cell viability was presented as a percentage of viable cells to control cells. Each experiment was performed three times.

### Western blot

Cell lysates were prepared and western blot analysis was performed as described previously [22]. Antibodies against the following proteins were used: phosphorylated EGFR (p-EGFR,Y1068, #2234), Total EGFR (T-EGFR, #2232),phosphorylated Signal transducer and activator of transcription 3(p-STAT3,Y705, #9145), Total STAT3 (T-STAT3, #4904), phosphorylated v-Akt Murine Thymoma Viral Oncogene (p-AKT, Ser473, #9271), Total AKT (T-AKT, #9272), phosphorylated Extracellular Signal-regulated Kinase (p-ERK, Thr202/Tyr204, #9101), Total ERK (T-ERK, #9102), heat shock protein 60(HSP 60, #12165), Vimentin(#5741), phosphorylated focal adhesion kinase (p-FAK, Y397, #3283), Total FAK(T-FAK, #3285), α-tubulin(#2144) (all antibodies except type I collagen were purchased from Cell Signalling Technology, Danvers, MA, USA, 1:1000). Type I collagen (abcam, Cambridge, UK, #34710). The band densitometric analysis was performed by using the public software Image J (Wayne Rasband National Institutes of Health, Bethesda, MD, USA). Expression of α-tubulin was used to correct for variation in sample loading.

### Transfection with short interfering RNA (siRNA)

All siRNA transfection experiments were performed using Lipofectamine 2000 reagent (Invitrogen, Carlsbad, USA) as described previously [20]. After incubation for 24 h, the medium was removed and the cells were washed with PBS and treated with NTP for 24 h. EGFR-, STAT3-specific, and control siRNAs were acquired from Genolution Pharmaceuticals (Seoul, Korea).

### Reverse transcription-polymerase chain reaction (RT-PCR)

Total RNA was extracted from homogenized cells using TRIzol reagent (Gibco-BRL, Grand Island, NY, USA). ReverTraAce qPCR RT master mix (Toyobo Co. Ltd., Osaka, Japan) was used to reverse-transcribe RNA. Total RNA (1 μg) was mixed with 10μg of the mixture. Reverse transcription was performed and cDNA was synthesized. The cDNA was added to a mixture of Quick Taq HS DyeMix (Toyobo Co. Ltd.) and specific primers, and amplified using a T100^™^ Thermal Cycler (Bio-Rad, Waltham, MA, USA). Reverse transcription was performed and cDNA was synthesized as described previously.[19] RNA primer sequences were as follows: Type I collagen -F, 5′-GGG CAA GAC AGT GAT TGA ATA-3′; Type I collagen -R, 5′-ACG TCF AAG CCG AAT TCC T-3′; Type III collagen-F, 5′-AGG TCC TGC GGG TAA CAC T-3′; Type III collagen -R, 5′-ACT TTC ACC CTT GAC ACC CTG-3′; TGF-β F, 5′-CCG ACT ACT ACG CCA AGG-3′; TGF-β R, 5′-AGT GAA CCC GTT GAT GTC CA-3′ and GAPDH-F, 5′-ACC ACA GTC CAT GCC ATC AC-3′; GAPDH-R, 5′-TCC ACC ACC CTG TTG CTG TA-3′. After denaturation for 3 min at 94°C, samples were amplified for 30 cycles of 30 s at 94°C, 62°C and 72°C, with a 5-min extension at 72°C. The products were separated by electrophoresis in 1.5% agarose gels and detected using ultraviolet light (Bio-Rad, Hercules, CA, USA).

### Sircol collagen assay

Total soluble collagen in cell culture supernatants was quantified using the Sircol collagen assay (Biocolor, Belfast, UK). For these experiments, confluent cells in 60 mm^2^ culture dishes were incubated for 24 h. One mL of Sirius red stain, an anionic dye that reacts specifically with basic collagen side chain groups, was added to 400 μL of supernatant and incubated with gentle rotation for 30 min at room temperature. After centrifugation at 12,000 g for 10 min, the collagen-bound dye was dissolved again after addition of 1 mL of 0.5 M NaOH and absorbance at 540 nm was measured by enzyme-linked immunosorbent assay (Bio-Tek). The absorbance was directly proportional to the amount of newly formed collagen in the cell culture supernatant.

### Immunofluorescence microscopy

Cells were cultured on microscope coverslips (Thermo Fisher Scientific, Rochester, NY, USA) and treated with NTP. After 24 h, the slides were washed with PBS, fixed for 20 min in 3.7% formaldehyde and rehydrated in PBS. After blocking for 45 min in bovine serum albumin dissolved in 5% PBS, the slides were incubated for 1 h with antibodies against p-STAT3 (Y705) (1:50; Cell Signaling Technology, Danvers, MA, USA, #9145) and Type I Collagen (1:50; Abcam, Cambridge, UK, #34710), and then washed with PBS and incubated for 45 min with an Alexa 488-labeled goat anti-rabbit antibody (1:250; Thermo Fisher, Eu- gene, OR, USA, # A-11034). After rinsing in PBS, Hoechst 33258 and phalloidin (1:50; Molecular Probes, Eu- gene, OR, USA, R415) were added for 15 min to counterstain nuclei and F-actin. The slides were washed with PBS, mounted with Vectashield (Vector Laboratories, Inc., Burlingame, CA, USA) and then analysed using fluorescence microscopy (Carl Zeiss, Germany).

### Immunohistochemistry

Immunohistochemical staining against p-STAT3 (Y705, 1:200; Cell Signaling Technology, Danvers, MA, USA, #9145), EGFR (1:50, Cell Signaling Technology, Danvers, MA, USA, #4267), Type I collagen (1:500, abcam, Cambridge, UK, #34710), Type III collagen(1:250, abcam, Cambridge, UK, #7778) was performed on paraffin-embedded tumor sections collected on poly-L-lysine-coated slides as described previously [22]. H&E (Hematoxylin and eosin) staining was performed as follows: hematoxylin staining (15 min, Room temperature) and hydrochloric acid alcohol solution for 35 s decoloring, eosin staining (10 min, Room temperature) and 90% ethanol for 40 s decoloring. Then neutral balsam was used for mounting and the section was observed and photographed under the microscope. Measurements of p-STAT3 positive cells were performed on 10–15 images per slide captured by an independent observer who was blind to the experimental condition.

### Statistical analysis

All of the data were derived from independent experiments, and the parameters are expressed as the means ± standard deviations. Statistical significance of differences between compared groups in each assay were analysed using Mann-Whitney U test, one-way analysis of variance and Tukey’s and least significant difference *post hoc* tests (SPSS, Chicago, IL, USA). Differences were considered statistically significant when *P* < 0.05 and illustrated as follows: * *P* < 0.05; ** *P* < 0.01; *** *P* < 0.001.

## Results

### NTP inhibited cell migration in KFs, but not in NFs

**Fig 1:**
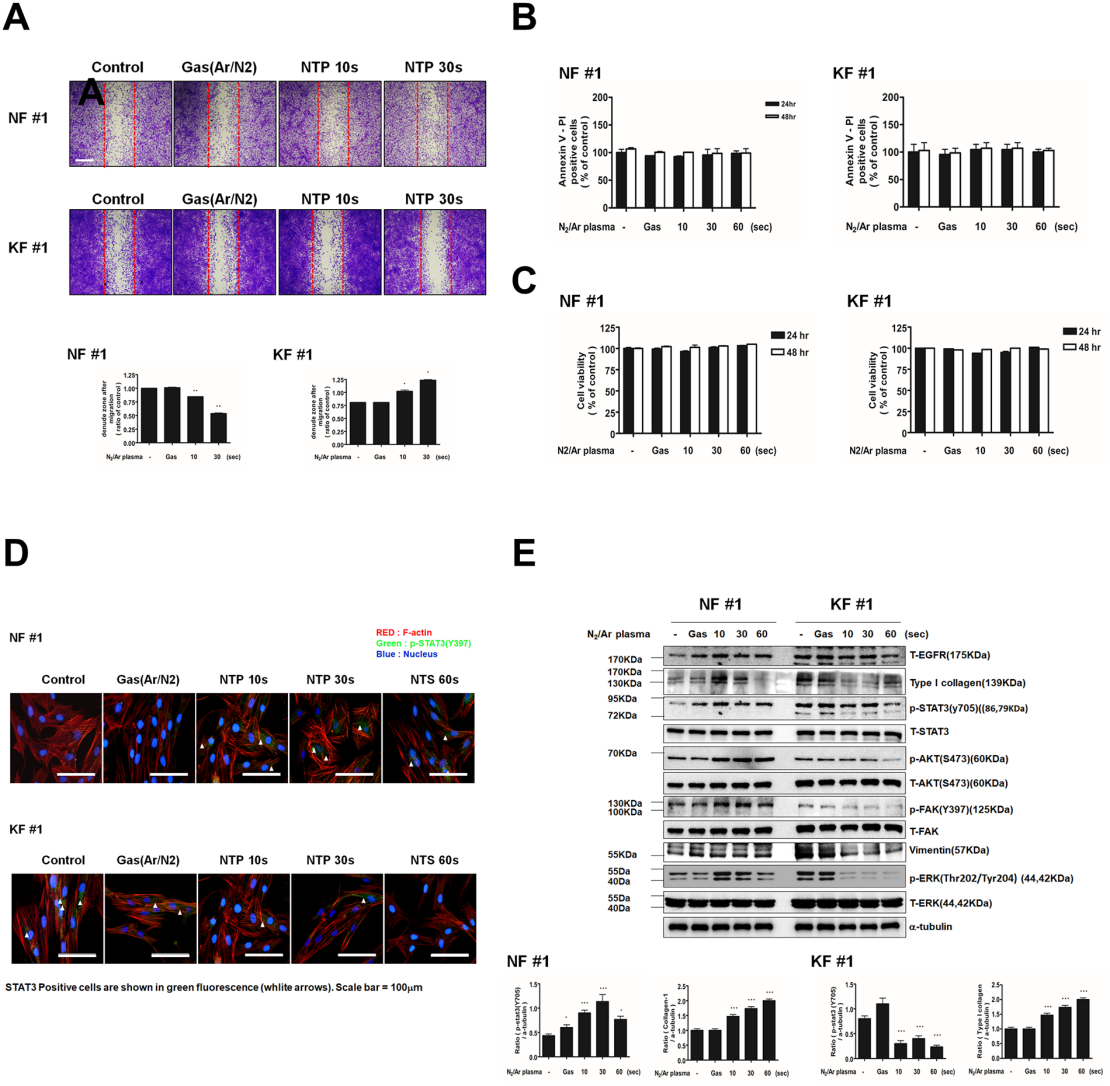
Effects of NTP treatment on cell migration, cell death, cell viability and protein expression in KFs and NFs. (A) Scratch wound healing assay after NTP treatment on NF and KF. Wound healing was documented by photography. (B) Annexin-V and propidium iodide staining in NFs and KFs. (C) MTT assay for cell viability of NFs and KFs. (D) Western blotting analysis for T-EGFR, Type I collagen, p-STAT3, T-STAT3, p-AKT, T-AKT, p-FAK, T-FAK, vimentin, p-ERK, T-ERK in NFs and KFs after NTP treatment. (E)Immunofluorescent staining of NFs and KFs was performed using p-STAT3 and F-actin antibody. DAPI was used for nuclei counter staining. After NTP exposures for 10, 30, or 60 s and after the control gas treatment.(Green = p-STAT3, RED = phalloidin, F-actin, Blue = DAPI). Data represent mean±standard deviation of three independent experiments. Each figure was representative of three experiments with triplicates. Scale bar = 100 μm. *P < .05, **P < .01 and ***P < .001.

To examine the effect of NTP treatment on cell migration, which is an important cellular mechanism in wound healing of NFs, as well as in KF-mediated keloid formation, wound healing assays were performed. As shown in Fig 1A, NTP meaningfully suppressed KF migration across the denuded zone (*P* < 0.001). In the graph, the denuded zone increased in KF, indicating the inhibitory effect of NTP. NTP treatment for 10 s and 30 s inhibited cellular migration in KFs by 3% and 36% compared to migration in control cells, respectively, after 24 h of incubation. In contrast, the same treatment enhanced cell migration in NFs by 4% and 28%, respectively.

To determine whether NTP induced cell death in NFs and KFs, annexin V/PI assay was performed. NTP treatment did not increase the percentage of annexin V/PI double positive cells, which indicates the absence of NTP-induced apoptosis in both NFs and KFs 24 h and 48 h after NTP treatment (Fig 1B). The viability of the cells after NTP treatment was measured by MTT assay and the result showed that NTP did not affect NFs or KFs viability (Fig 1C).

### NTP decreased EGFR, STAT3 and Type 1 collagen in KFs, whereas opposite effects of NTP were observed in NFs

Previous studies have reported that activation of several intracellular signalling cascades such as smad3, mitogen-activated protein kinases and STAT3 induces abnormal biological properties of keloid tissue [6, 23–27]. Thus, we examined which signal pathways are involved in the NTP-induced suppression of cell migration in KFs and enhancement of cell migration in NFs. In 24 h after NTP treatment for 10, 30s or 1 min, levels of T-EGFR, p-STAT3, p-ERK, and Type I collagen were analysed by western blot (Fig 1D). A considerable decrease of EGFR and p-ERK was observed in KFs after NTP treatment, whereas their expressions in NFs remained relatively constant. Interestingly, NTP treatment increased p-STAT3 and p-AKT level and Type I collagen production in NFs, but inhibited them in KFs. In addition, previous report demonstrated that FAK is involved in cell migration through FAK/Src complex, which is a down-stream molecule of EGFR signalling [28]. Thus, we investigated the effect of NTP on p-FAK level. Treatment with NTP increased p-FAK in NF, but decreased the level in KF. To verify that the decrease in p-STAT3 level, immuno-fluorescence microscopy was performed using an anti-p-STAT3 antibody. NTP treatment suppressed p-STAT3 level in the KFs but enhanced its level in NFs, which is consistent with the results of western blot analysis (Fig 1E). Taken together, these results suggest that NTP suppresses T-EGFR, p-STAT3, and p-ERK, p-AKT signalling pathways in KF and that these suppressions are correlated with KF cell migration.

### EGFR and STAT3 expression in NS and KS are relatively different

**Fig 2:**
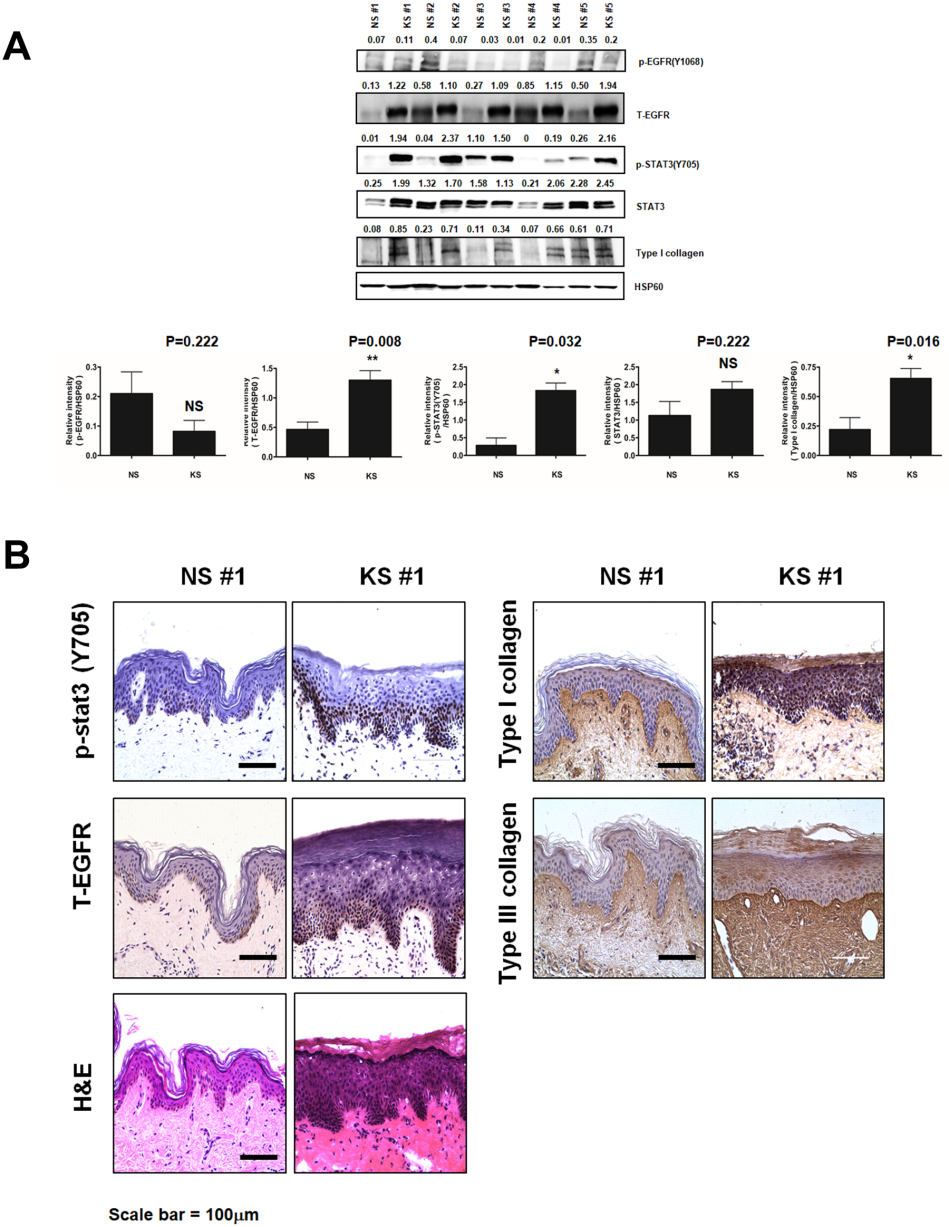
Comparisons of protein expression and protein phosphorylation in NFs and KFs. (A) The expression levels of p-EGFR, T-EGFR, T-STAT3, p-STAT3, and Type I collagen in NS and KF tissues were compared. Quantitative analysis of expression levels were made in NS (5 cases) and KF (5 cases). The expression of is EGFR, p-STAT3 and Type I collagen increased in KF tissues compared with NS. (B) Haematoxylin-eosin staining and immunohistochemical staining with antibodies against p-STAT3, T-EGFR, and Type I collagen and III were performed in paraffin sections of normal and keloid skin tissue specimens from patients. Data represent mean±standard deviation of three independent experiments. Each figure was representative of three experiments with triplicates. Scale bar = 100 μm. *P < .05, **P < .01 and ***P < .001.

To determine if similar changes could be observed *in vivo*, tissue lysates of five NS and five KS samples from patients have been investigated. NS samples showed low levels of p-STAT3 and EGFR (Fig 2A). In contrast, KS lysates showed moderate to high level of p-STAT3 and high level of T-EGFR. Type I collagen production was also elevated in KS lysates, whereas only one of the five NS lysates showed elevation of collagen production. These results were confirmed by immunohistochemistry. Immunohistochemical staining indicated that KS samples had a stronger expression of p-STAT3, T-EGFR, and Type I and III collagen than NS specimens (Fig 2B).

### Inhibition of EGFR and STAT3 by siRNA suppressed cell migration in KFs and NFs

**Fig 3:**
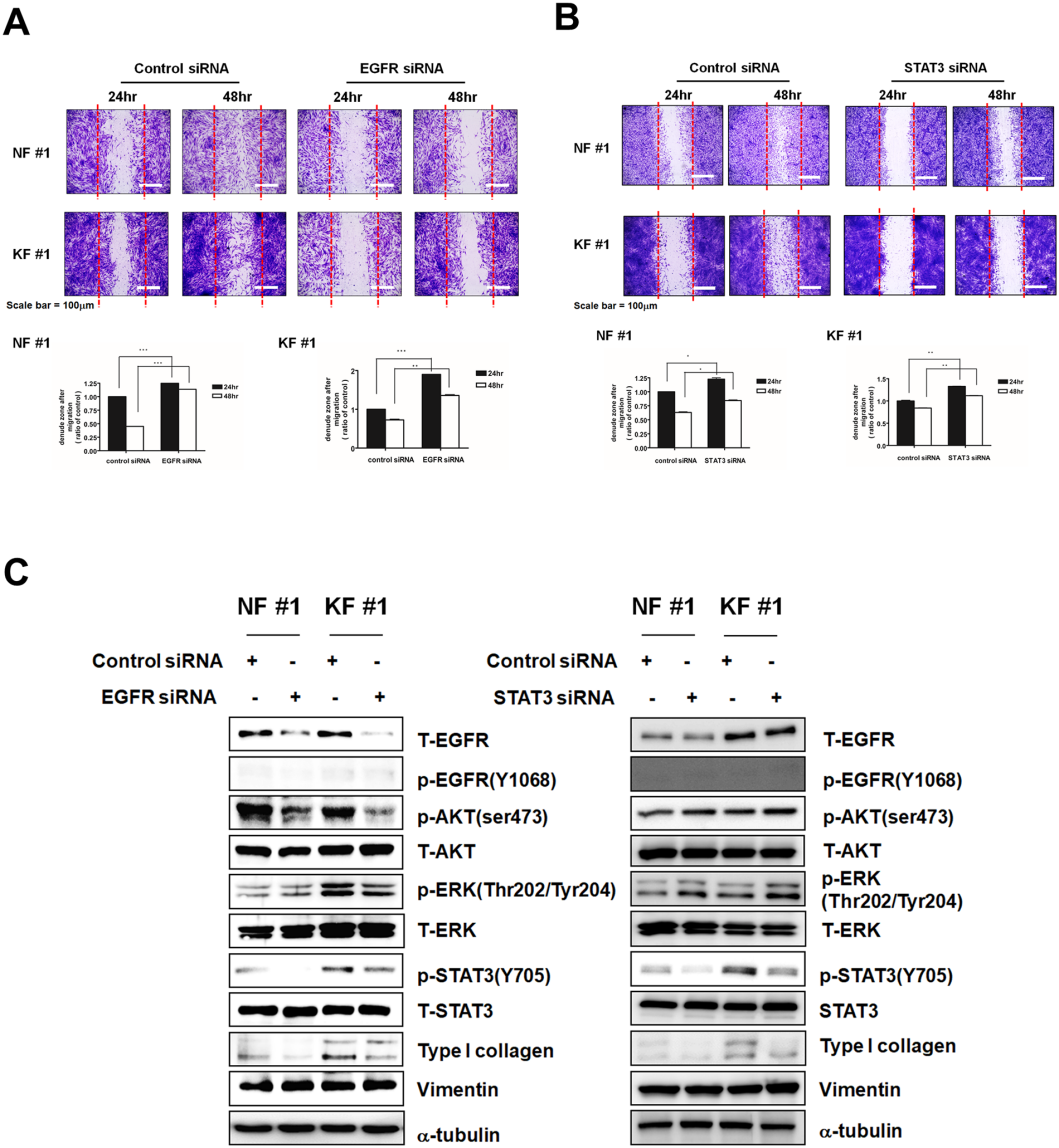
Effects of EGFR and STAT3 specific siRNAs on NFs and KFs. (A) Migration of NFs and KFs was assessed by the wound healing assay. Cell migration of NFs and KFs was lower after 24 and 48h treatments with EGFR-specific rather than control siRNA. (B) STAT3 siRNA had a higher inhibitory effect on cell migration in NFs and KFs than control siRNA after 24 and 48h treatments. (C) Western blotting for T-EGFR, p-EGFR, p-AKT, T-AKT, p-ERK, T-ERK, p-STAT3, T-STAT3, Type I collagen, vimentin, and α-tubulin in NFs and KFs after treatment with EGFR and STAT3 specific siRNAs. Data represent mean±standard deviation of three independent experiments. Each figure was representative of three experiments with triplicates. Scale bar = 100 μm. *P < .05, **P < .01 and ***P < .001.

To evaluate the role of EGFR and STAT3 in cell migration, we conducted the wound healing assay after treating KFs and NFs with EGFR-specific, STAT3-specific or control siRNAs for 24 h or 48 h. As observed in Fig 3A, cell migration was efficiently suppressed by the treatment with EGFR-specific siRNA than control siRNA both in NFs and KFs. Similarly, the inhibition of STAT3 expression by a STAT3-specific siRNA inhibited cell migration both in NFs and KFs (Fig 3B).

These findings suggest that EGFR and STAT3 inhibition may be a major mechanism in the suppression of cell migration by NTP. After wound healing assays, we also found that the treatment with EGFR-specific siRNA decreased the expression of T-EGFR, p-AKT, p-STAT3, and Type I collagen in NFs and KFs by western blot analysis. Taken together, these data demonstrated that EGFR and STAT3 are important signal mediator of cell migration in fibroblast, and the treatment with NTP inhibited KFs cell migration via decreasing the activity of the EGFR/STAT3 pathway (Fig 3C).

### NTP suppresses collagen production in KFs by suppressing TGF-β expression but has no effect on NFs

**Fig 4:**
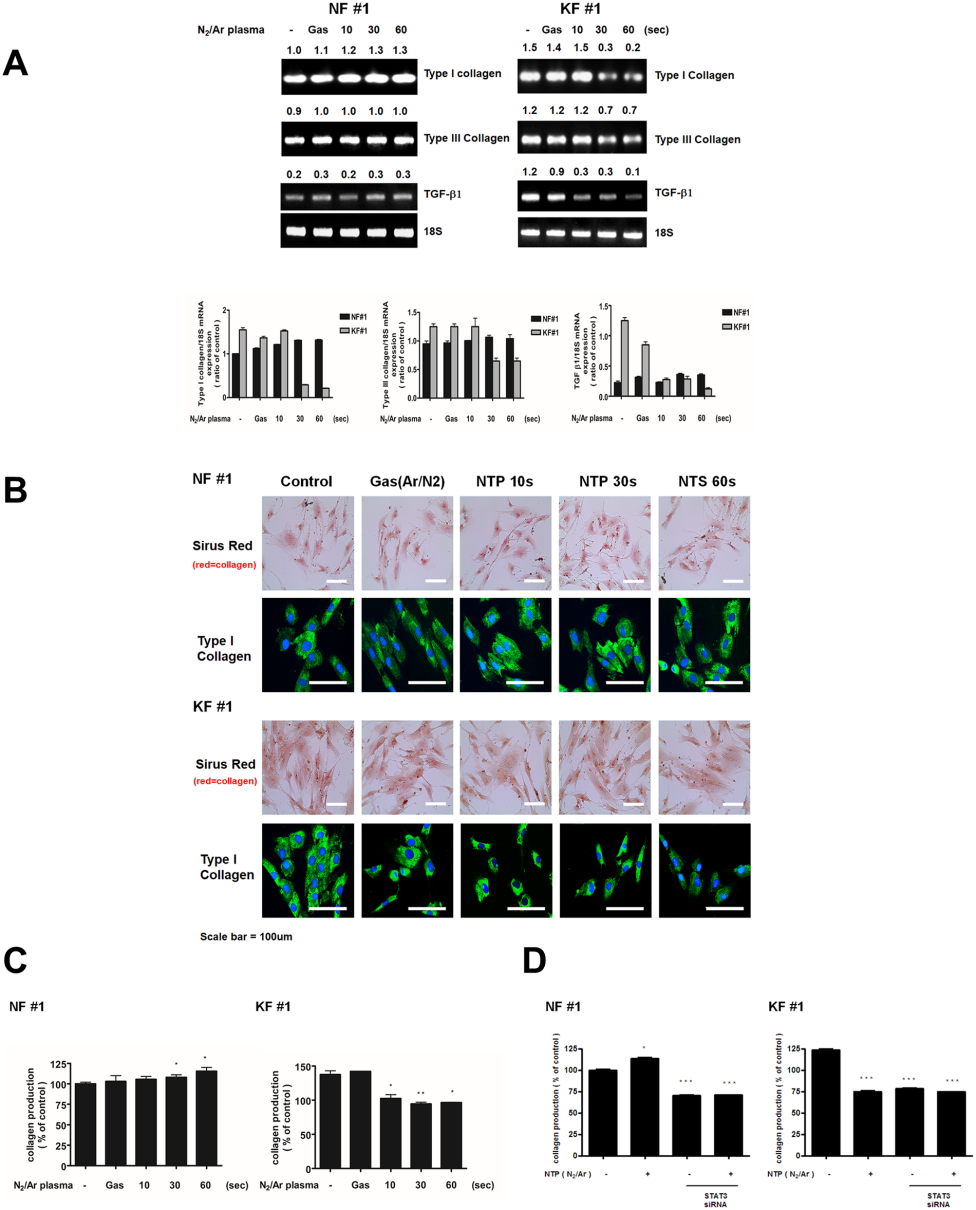
Collagen production after NTP treatment in NFs and KFs. (A) Reductions in Type I collagen, Type III collagen, and TGF-β mRNA expression evaluated by RT-PCR in KFs that were exposed to NTP for 10, 30, and 60 s as well as to control gas treatment. (B) NTP reduced the expression of Type I collagen in KFs as revealed by the immunocytochemistry and Sirus Red stain. Immunofluorescent and Sirus Red staining for Type I collagen were observed in NFs and KFs after treatments with gas or NTP. (C) Total soluble collagen were measured by Sircol collagen assay kit after treatment with NTP in NF and KF. (D) Collagen production levels were measured after combination treatment with NTP and STAT3 siRNA in NF and KF. Data represent mean±standard deviation of three independent experiments. Each figure was representative of three experiments with triplicates. Scale bar = 100 μm. *P < .05, **P < .01 and ***P < .001.

Increased collagen deposition mediated by enhanced TGF-β signalling represents the main mechanism of keloid formation [10, 29]. We therefore assessed expression levels of Type I collagen, Type III collagen, and TGF-β in NFs and KFs by RT-PCR (Fig 4A). No significant changes in Type I collagen, Type III collagen, and TGF-β mRNAs were observed following NTP treatment in NFs. However, in KFs, NTP treatment comparatively suppressed expression of all these three mRNAs. Using the Sircol collagen assay, we found that NTP reduced soluble collagen content in KFs, while the treatment slightly increased the total soluble collagen amount in NFs (Fig 4C). This was also confirmed by immunofluorescent staining for Type I collagen (Fig 4B). The effect of single treatment with NTP or STAT3 siRNA on reduced production of total collagen in KFs was similar to that of combination treatment with NTP and STAT3 siRNA (Fig 4D). Consistently, it is also known that inhibition of STAT3 expression suppresses collagen production [26]. These results suggest that the suppression of collagen production by NTP treatment in KFs is through the down-regulation of STAT3 activation.

## Discussion

Keloid pathogenesis involves anomalous fibroblast activity including perturbed proliferation, migration, and collagen synthesis. However, the detailed mechanisms of keloid development still remain unknown and even the most effective treatment for keloid remains controversial. Although several therapeutic strategies, such as surgery combined with radiotherapy [3] or intralesional steroid injection [7, 30], are used to suppress the uncontrolled keloid activity, none is fully effective. Therefore, it is crucial to understand the pathogenesis of keloids in order to develop superior treatment approaches.

NTP is a rapidly growing new research area in health care. One promising novel medical application of NTP is a cancer treatment, but NTP also has different healing effects on normal cells. Our group previously showed that NTP has not only an anti-cancer effect [19, 22, 31] but also an effect on muscle regeneration [32] and wound healing [18]. In this study, we found that NTP treatment suppressed KF cell migration, but increased it in NFs *in vitro*. Cell migration is a main process of wound healing and is also important for keloid formation. KF shows a higher migration pattern toward the midline of a scratch wound in comparison with NF [26]. Thus, suppression of KF activation has been proposed as a therapeutic strategy for the treatment and prevention of keloids [33]. In this study, we demonstrated that NTP treatment could regulate cell migration of KF and NF, and could have an effect on supressing keloid formation and enhancing NF wound healing.

After ensuring the regulatory effect of NTP on the cell migration, we investigated whether NTP could be used for treatment of KF. To elucidate the mechanism of the inhibitory effect of NTP on keloid, we compared signalling responses of NFs and KFs. The result showed that T-EGFR, p-STAT3 and Type I collagen levels were higher in KF than NF and the treatment with NTP decreased the levels of T-EGFR, p-STAT3 and Type I collagen in KF. However, the treatment with NTP increased these levels in NF.

Satish *et al*. revealed that KFs show altered responses to various growth factors, including the EGF, which influences multiple processes including cell migration, cell proliferation, matrix production and degradation [6]. STAT3 is activated by various growth factors and contributes to keloid pathogenesis by promoting collagen production, cell proliferation, and migration [26]. As reported by Lim *et al*., STAT3 is associated with cytokines that increase KF proliferation, suggesting that altered cytokine balance is involved in KF pathogenesis [5]. Our results are in agreement with these earlier studies because we found that NTP inhibited cell migration by suppressing EGFR expression and STAT3 activation in KFs, whereas it had opposite effects in NFs (Fig 2D).

A lot of studies have indicated that TGF-β may induce keloid formation [9, 27, 34, 35]. TGF-β is a multifunctional cytokine that regulates cell growth, differentiation, and biosynthesis of extracellular connective tissue. It also stimulates collagen synthesis [36, 37] and inhibits protease production [38]. KFs produce excessive amounts of collagen and up-regulation of TGF-β positively influences proliferation of KFs and collagen overproduction. In this study, using sirius red staining, we found that NTP treatment decreased collagen levels in the extracellular matrix of keloid tissues. We also observed that NTP reduced TGF-β expression in KFs, implying that NTP could be a physiological TGF-β inhibitor, which interacts with collagen, and influences collagen synthesis resulting suppression of keloid formation.

Our results demonstrated that NTP down-regulates the EGFR/STAT3 pathway, which suppresses TGF-β expression and thereby decreases KF cell migration and collagen production. Inhibition of EGFR/STAT3 pathway by NTP may be a useful therapeutic and preventive strategy for keloid therapy. On the other hand, NTP treatment up-regulated EGFR/STAT3 pathway and collagen production as well as increased cell migration in NFs. NTP can therefore have beneficial therapeutic effects mediated by the EGFR/STAT3 signalling pathway on the pathogenesis of keloid scars and on normal wound healing.

To the best of our knowledge, this study is the first to document opposite paradoxical effects of NTP on NFs and KFs. Although this study is very limited, but it is still a meaningful result and it would be beneficial to many keloid patients if they are actually used clinically after animal experiments and clinical trials, our findings imply that NTP has a therapeutic potential to intervene keloids.

## Supporting information

S1 Fig A schema of the experimental system. (TIF)

S2 Fig Annexin-V and propidium iodide staining in NF#1 and KF#1 after 10s, 30s and 60s NTP treatment. (TIF)

S3 Fig Western blotting band intensities were measured and represented as a graph for EGFR, p-AKT, p-FAK, vimentin, p-ERK in NF#1 and KF#1 after 10s, 30s and 60s NTP treatment. (TIF)
