# Supplementary figures and images for: Opposite effects of non-thermal plasma on cell migration and collagen production in keloid and normal fibroblasts
Source: PLoS One. 2017 Nov 16;12(11):e0187978. doi: 10.1371/journal.pone.0187978 (PMC5690474; doi:10.1371/journal.pone.0187978)

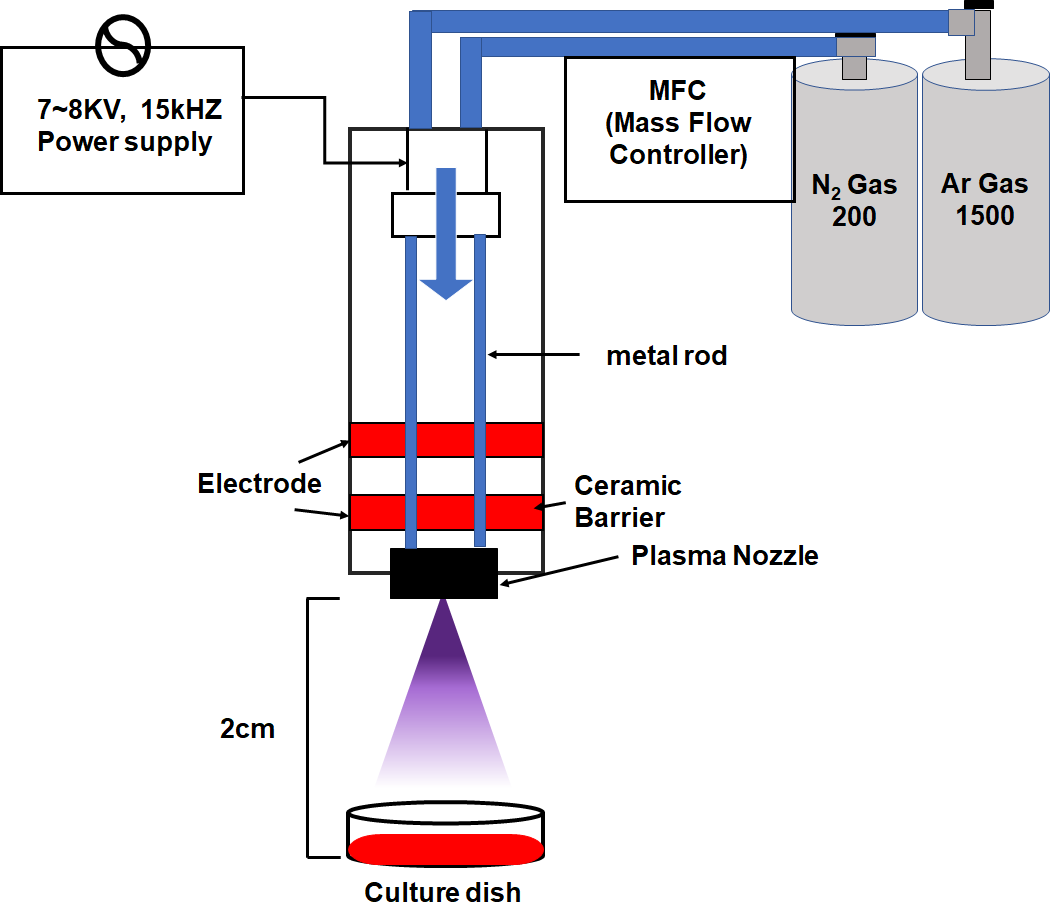

Supplement: S1 Fig — (TIF) [file pone.0187978.s001.tif]

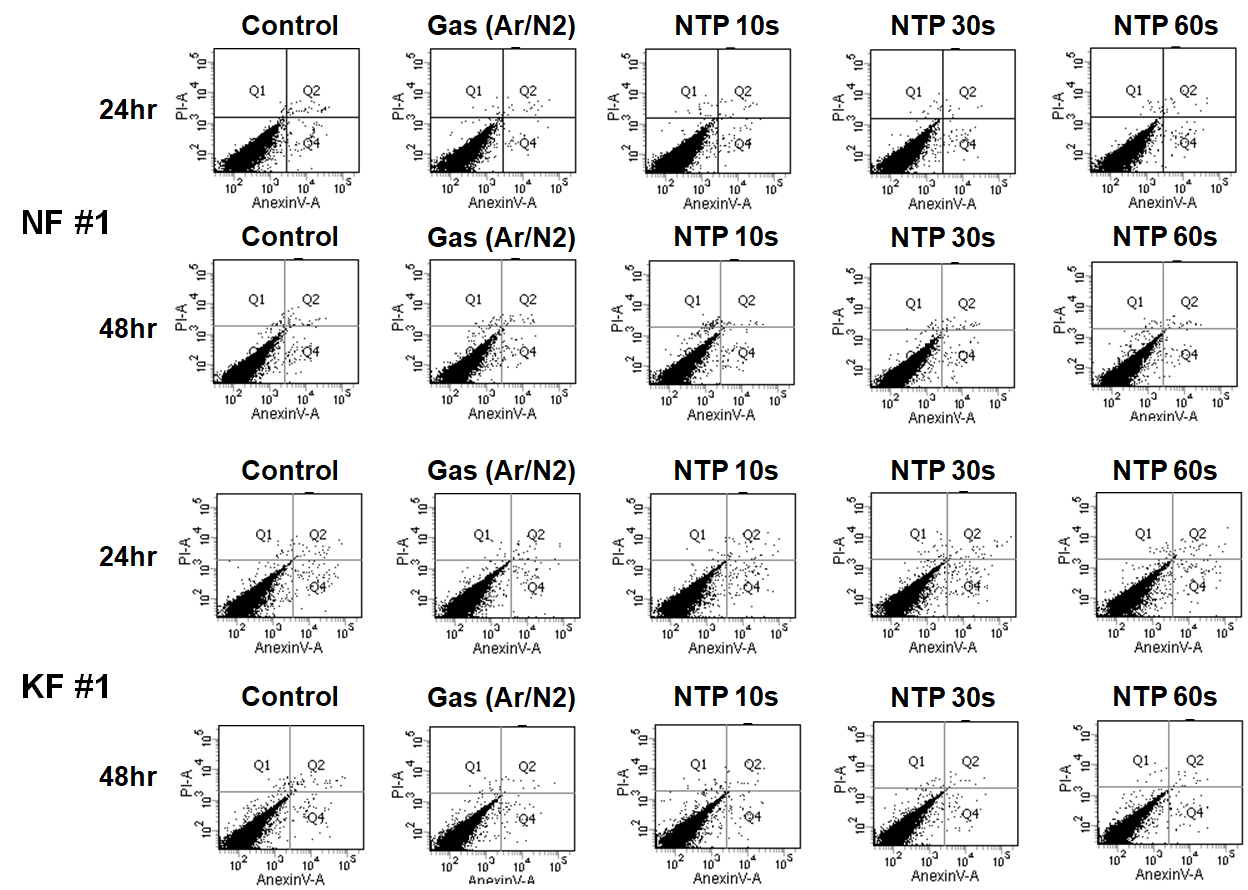

Supplement: S2 Fig — (TIF) [file pone.0187978.s002.tif]

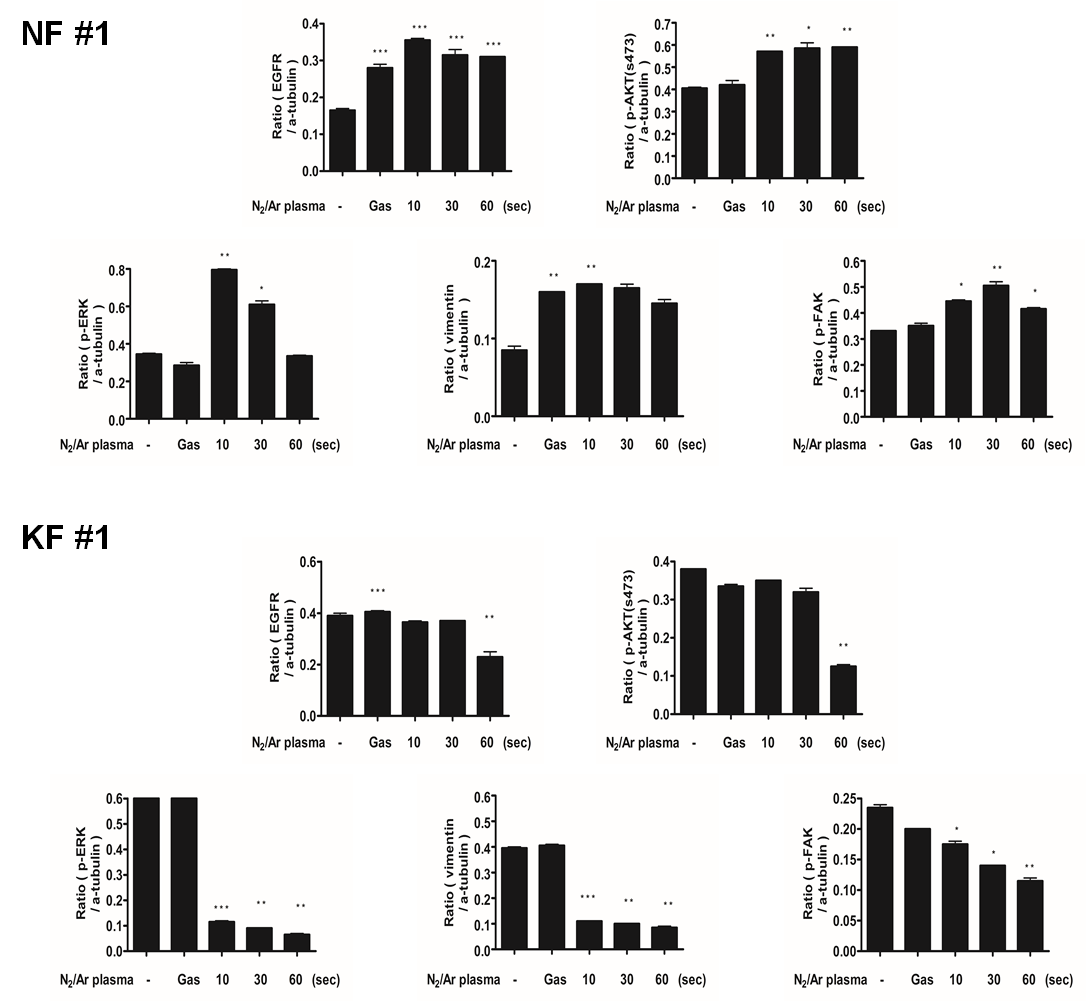

Supplement: S3 Fig — (TIF) [file pone.0187978.s003.tif]
